# Gut Microbiota Dysbiosis in Childhood Vasculitis: A Perspective Comparative Pilot Study

**DOI:** 10.3390/jpm12060973

**Published:** 2022-06-15

**Authors:** Marianna Fabi, Federica D’Amico, Silvia Turroni, Laura Andreozzi, Emanuele Filice, Patrizia Brigidi, Marcello Lanari

**Affiliations:** 1 Pediatric Emergency Unit, Department of Medical and Surgical Sciences, IRCCS Azienda Ospedaliero-Universitaria, University of Bologna, Via Massarenti 9, 40138 Bologna, Italy; marianna.fabi@aosp.bo.it (M.F.); emanuele.filice@studio.unibo.it (E.F.); marcello.lanari@unibo.it (M.L.); 2 Microbiomics Unit, Department of Medical and Surgical Sciences, University of Bologna, Via Massarenti 9, 40138 Bologna, Italy; federica.damico8@unibo.it (F.D.); patrizia.brigidi@unibo.it (P.B.); 3 Unit of Microbiome Science and Biotechnology, Department of Pharmacy and Biotechnology, University of Bologna, Via Belmeloro 6, 40126 Bologna, Italy

**Keywords:** Kawasaki disease, Henoch–Schönlein purpura, gut microbiota, vasculitis, intestinal inflammation, dysbiosis, abdominal involvement, children

## Abstract

Kawasaki disease (KD) and Henoch–Schönlein purpura (HSP) are the most frequent vasculitis in childhood. For both, a multifactorial mechanism has been hypothesised, with an abnormal immune response in genetically predisposed children. Gut microbiota (GM) alterations might trigger the hyperimmune reaction. Our aim was to explore the GM in KD and compare it with the GM of HSP and febrile children. Children diagnosed with KD, HSP and non-KD febrile illness (F) were enrolled. GM was profiled by 16S rRNA gene sequencing and compared with the profiles of healthy children from previous studies. We enrolled 13 KD, 10 HSP and 12 F children. Their GM significantly differed from controls, with an overall reduction in the relative abundance of beneficial taxa belonging to the *Ruminococcaceae* and *Lachnospiraceae* families. Potential KD and HSP signatures were identified, including smaller amounts of *Dialister* in the former, and *Clostridium* and *Akkermansia* in the latter. Notably, the GM structures of KD, HSP and F patients stratified by abdominal involvement, with more severe dysbiosis in those suffering from intestinal symptoms. This is the first study analysing GM in a mostly Caucasian cohort of KD and HSP children. Our data could open up new opportunities for childhood vasculitis treatment.

## 1. Introduction

Childhood vasculitis is a group of conditions characterised by inflammation of the vascular wall, leading to multisystemic involvement and a variety of clinical manifestations. Diagnosis may be challenging and often requires the cooperation of different specialists [1]. In childhood, the most common primary vasculitis are Henoch–Schönlein purpura (HSP) and Kawasaki disease (KD), accounting for 49% and 23% of all cases, respectively [2].

HSP is a small vessel vasculitis, characterised by the deposition of immunoglobulin A (IgA)-containing immune complexes in the vessel walls. HSP typically involves the skin, gut, kidney and joints, resulting in palpable purpura, arthralgia and/or arthritis, gastrointestinal symptoms and glomerulonephritis [3,4]. Although the histological features are widely known (leukocytoclastic vasculitis with predominant IgA deposit), its aetiology is still unclear [3,4]. Since HSP often occurs in children with a history of upper respiratory tract infections during the previous 2–4 weeks, a dysregulated immune response to an infectious trigger has been proposed as a possible etiologic basis [3].

On the other hand, KD is an acute systemic vasculitis affecting small and medium-sized arteries, which predominantly occurs in children aged less than 5 years. It is the leading cause of acquired heart disease in developed countries, due to the occurrence of coronary artery lesions in ~4% of cases, and subsequent severe complications, such as ischemic heart disease and sudden death during the acute and subacute stages of the illness [5].

Although KD was firstly described in 1967 by Dr. Tomisaku Kawasaki, its aetiology is also not yet precisely defined: clinical and epidemiological features suggest that an environmental agent, probably a virus [6,7,8], triggers an abnormal immune response in a genetically susceptible child [9,10]. The genetic background is strongly supported by the incidence that differs considerably among ethnicities, being the highest in Asian children and Asian descendants in the transmigration area [11,12,13].

Hence, KD and HSP share a similar pathway where an immune-mediated mechanism is triggered by an environmental agent, leading to a variety of clinical manifestations in genetically predisposed children [14]. KD and HSP both potentially affect intestinal vessels: indeed, abdominal involvement can occur in up to one-third of KD patients and up to 50% of HSP patients [4,15].

In recent decades, the gut microbiota (GM), i.e., the complex and diverse community of trillions of primarily bacterial cells that populate our gastrointestinal tract, has been extensively studied for its role in regulating several aspects of human physiology, including immune homeostasis [16]. It has recently become obvious that GM alterations can cause immune dysregulation, contributing to a plethora of disorders, both intestinal and systemic, such as inflammatory bowel, allergic and autoimmune diseases [17,18,19,20,21]. Regarding childhood vasculitis, a number of studies have explored GM in Asian KD and HSP cohorts, highlighting potentially common features, such as reduced microbial diversity (common marker of dysbiosis) and reduced proportions of health-associated and anti-inflammatory taxa (e.g., *Lachnospiraceae* and *Ruminococcaceae* members) compared to healthy subjects [22,23,24,25]. The loss of GM homeostasis was also corroborated by the increase in pathobionts (mainly *Enterococcus*) in both KD and HSP [24,25]. However, none of these studies directly compared different forms of vasculitis and, to our knowledge, no data are currently available on other populations of different ethnicities, although the latter plays a key role in the predisposition to immune-mediated diseases [12,13,26] and is closely associated with GM structure [27].

In an attempt to fill this gap, here we characterised, through 16S rRNA gene sequencing, the GM composition of KD and HSP patients in a mostly Caucasian paediatric population, as compared to non-KD febrile children (F) and healthy controls (HC). The GM profile of patients was then correlated with clinical features and laboratory data, particularly with markers of disease activity and inflammation. Finally, since abdominal involvement is frequent in all these diseases and it has been found as a possible risk factor for severe coronary artery lesions (CALs) in KD [15], associations between GM structure and abdominal symptoms were sought as well.

## 2. Materials and Methods

### 2.1. Study Design, Patients and Sample Collection

A monocentric prospective pilot study was conducted on paediatric patients diagnosed with KD, HSP or non-KD febrile illness at the S. Orsola-Malpighi Hospital, University of Bologna (Bologna, Italy), aged 0–14 years, from July 2017 to November 2019.

All KD diagnoses were made in accordance with 2017 American Heart Association (AHA) guidelines [5]. The onset of illness was defined as the first day of fever. Standard treatment consisted of intravenous immunoglobulin (IVIG) at 2 g/kg in a single infusion before the 10th day of fever, together with aspirin at 30–50 mg/kg/day, subsequently switched to 3–5 mg/kg/day once the patient became afebrile for at least 48 h. Complete and incomplete forms were defined according to 2017 AHA criteria. IVIG resistance was defined as persistent or recrudescent fever at least 36 h after the end of IVIG infusion.

Diagnosis of HSP was made in accordance with EULAR/PRINTO/PRES criteria [4]. Corticosteroid therapy was administered when appropriate (severe gastrointestinal or renal involvement).

The coronary artery (CA) status of KD subjects was assessed by echocardiography during the acute and subacute phase. Measurements of the internal diameters of the proximal right CA (RCA) and left anterior descending (LAD) CA were normalised for body surface area and expressed as standard deviation units from the mean (Z scores). CA was normal for Z score <2. CALs were defined as any kind of coronary involvement, i.e., dilation for Z score >2 and <2.5, aneurysms when >2.5. The Z score for either RCA or LAD at any time point or the Z score of the largest aneurysm was used for the continuous variable analysis. Cardiac non-coronary involvement was considered when echographic signs of ventricular dysfunction, endocarditis and pericarditis were documented during the acute stage of KD.

Gastrointestinal manifestations, such as vomiting, diarrhoea, abdominal pain, paralytic ileus, jaundice, pancreatitis and pseudo-obstruction, were considered according to the definitions previously described [15].

Exclusion criteria were confirmed bacterial or viral gastroenteritis, immunodeficiency and rheumatological and immunological disorders.

For comparative purposes, age- and sex-similar non-KD febrile subjects (F) were recruited from the emergency department if they had >3 days of fever.

Clinical data including age, ethnicity, gender, pre-treatment laboratory values and the presence of abdominal symptoms were recorded.

Blood and faecal samples were collected during the acute stage from each KD, HSP or F child, before the administration of disease-specific therapeutic approaches. Laboratory values included white blood cell count (WBC), neutrophil and lymphocyte percentage, red blood cell count (RBC), Haemoglobin (Hb), platelets (PLT), C-reactive protein (CRP), alanine aminotransferase (ALT) and aspartate aminotransferase (AST), immunoglobulin (Ig) G, IgA and IgM. Cytokine panels including tumor necrosis factor (TNF)-alpha, interleukin (IL) 6, IL8, IL10 and IL12p70 and faecal calprotectin were measured only in KD patients. Stool samples were sent to the Dept. of Pharmacy and Biotechnology (University of Bologna), where they were stored at −80 °C until processing for GM analysis.

The study was conducted in accordance with the Declaration of Helsinki, and the protocol was approved by the local Ethics Committee (Comitato Etico Area Vasta Emilia Centro, AVEC; project identification code: 178/2021/Sper/AOUBo). Special protections for children as research subjects were observed, according to local IRB guidelines. Written informed consent was provided by all parents or legal guardians.

### 2.2. Microbial DNA Extraction, Library Preparation and Sequencing

Microbial DNA was extracted from faeces using the repeated bead-beating plus column method, as previously described [28]. In short, about 250 mg of faecal sample was resuspended in 1 mL of lysis buffer (500 mM NaCl, 50 mM Tris-HCl pH 8, 50 mM EDTA, 4% SDS) and homogenised in a FastPrep instrument (MP Biomedicals, Irvine, CA, USA) at 5.5 movements/s for 1 min, repeated three times, in the presence of four 3 mm glass beads and 0.5 g of 0.1 mm zirconia beads (BioSpec Products, Bartlesville, OK). After 15 min incubation at 95 °C, stool particles were pelleted at 13,000 rpm for 5 min. The supernatant was added with 260 μL of 10 M ammonium acetate, incubated on ice for 5 min and further centrifuged at 13,000 rpm for 10 min. One volume of isopropanol was added to each sample and incubated on ice for 30 min. Nucleic acids were washed with 70% ethanol, then resuspended in TE buffer (10 mM Tris-HCl, 1 mM EDTA pH 8.0). After treatment with 2 μL of 10 mg/mL DNase-free RNase at 37 °C for 15 min, samples were subjected to protein removal and column-based DNA purification using the DNeasy Blood and Tissue Kit (QIAGEN, Hilden, Germany).

For library preparation, the V3-V4 hypervariable region of the 16S rRNA gene was amplified using the 341F and 785R primers with added Illumina adapter overhang sequences, as previously reported [29]. PCR products were purified using a magnetic bead-based clean-up system (Agencourt AMPure XP; Beckman Coulter, Brea, CA, USA). Indexed libraries were prepared by limited-cycle PCR using Nextera technology, and further cleaned up as described above. Final libraries were pooled at equimolar concentration (4 nM), denatured with 0.2 N NaOH and diluted to 5 pM with a 20% PhiX control before sequencing on an Illumina MiSeq platform, with a 2 × 250 bp paired-end protocol according to the manufacturer’s instructions (Illumina, San Diego, CA, USA). Sequencing reads were deposited in the National Center for Biotechnology Information Sequence Read Archive (NCBI SRA; BioProject ID PRJNA807281).

### 2.3. Bioinformatics and Statistics

Raw sequences were processed using a combined pipeline of PANDASeq [30] and QIIME 2 [31]. After length and quality filtering, reads were clustered into amplicon sequence variants (ASVs) using DADA2 [32]. Taxonomy was assigned using VSEARCH [33] against the Greengenes database (May 2013 release). Chimeras were discarded. The 16S rRNA gene sequencing data were compared with those of 35 healthy Caucasian children with similar age (mean ± SD, 5.8 ± 4.5 years) and male/female ratio (26 males, 9 females) as controls, from the following previous studies: (i) Rampelli et al. [34] (24 subjects, MG-RAST ID mgp84098); (ii) Muleviciene et al. [35] (10 subjects); and (iii) Biagi et al. [36] (1 subject, MG-RAST ID mgp17761). These control samples had been processed in the same laboratory as the study samples for DNA extraction, library preparation, sequencing and bioinformatics, thereby limiting study-related bias. Genus-level community composition was generated for combined cohorts. Alpha diversity was computed using the inverse Simpson index. Beta diversity was estimated by computing Bray–Curtis distances between the genus-level profiles, which were used as input for principal coordinates analysis (PCoA). All statistical analysis was performed in R 3.6.1. PCoA plots were generated using the vegan (http://www.cran.r-project.org/package-vegan/ (accessed on 2 April 2022)) and Made4 [37] packages, and data separation was tested by a permutation test with pseudo-F ratios (the “Adonis” function in vegan). Group differences in alpha diversity and taxon relative abundance were assessed by a Kruskal–Wallis test followed by a post hoc Wilcoxon test, as needed. *p* values were corrected for multiple comparisons using the Benjamini–Hochberg or false discovery rate (FDR) method. The Kendall rank correlation test was used to assess associations between genus-level relative abundances and inflammatory/immunological variables.

Demographic, clinical and laboratory data were reported as number and relative percentages if categorical, whereas continuous variables were presented as mean and standard deviation (SD) if normally distributed or as median and interquartile range (IQR) if not normally distributed. The Kolmogorov–Smirnov test was used to test for normality. Differences between groups were compared using ANOVA, Kruskal–Wallis or Mann–Whitney, as appropriate, for continuous variables and the Chi-squared test or Fisher’s exact test, as appropriate, for categorical variables, with post hoc comparisons (Fisher’s least significant difference or Dunn’s test) as appropriate. Levene’s test was used to assess the equality of variances. A *p* value ≤ 0.05 was considered statistically significant; a *p* value between 0.05 and 0.1 was considered a tendency.

## 3. Results

### 3.1. Study Cohort Description

A total of 35 samples were collected from 13 patients diagnosed with KD, 10 patients diagnosed with HSP and 12 F patients. Demographic and clinical data and pre-treatment laboratory values are displayed in Table 1.

Twenty-two patients (62.9%) were males, and the most frequent ethnicity was Caucasian (26 patients, 74.3%). The median age of all children was 40 months (IQR 19–62 months). Patients diagnosed with HSP were significantly older than KD and F patients (*p* = 0.005). Hb (*p* = 0.034), PLT (*p* = 0.010), CRP (*p* = 0.002), ALT (*p* = 0.011), IL6 (*p* = 0.025) and IgA (*p* = 0.031) were significantly different among the three groups. A post-hoc analysis showed that KD patients had significantly lower Hb (*p* = 0.029) and IgA (*p* = 0.026), and higher CRP (*p* < 0.001) and ALT (*p* = 0.008) compared to HSP patients, while they had higher PLT and IL6 (*p* = 0.037 and *p* = 0.025, respectively) compared to F patients. HSP patients had lower CRP (*p* = 0.027) and higher IgA levels (*p* = 0.017) compared to F patients. Notably, IgA was higher in HSP than in other groups, advocating its central role in the pathogenesis of HSP.

### 3.2. The Gut Microbiota Dysbiosis of KD, HSP and F Children

The GM of KD, HSP and F children was profiled by 16S rRNA gene sequencing and compared with that of healthy controls from previous studies [34,35,36] (see Materials and Methods). A total of 1,033,918 high-quality reads (mean ± SD, 29,540 ± 6439) were obtained, binned into 2108 ASVs.

No differences in alpha diversity were observed among study groups (*p* = 0.7, Kruskal–Wallis test) (Supplementary Figure S1).

**Figure 1:**
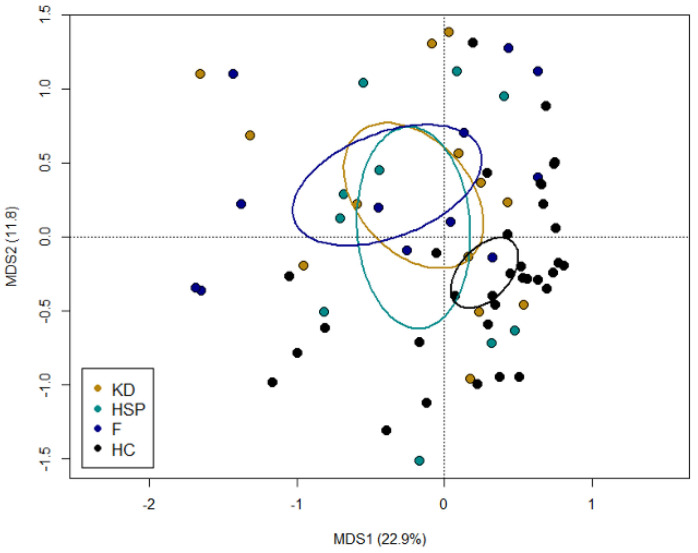
The gut microbiota of patients with Kawasaki disease (KD), Henoch–Schönlein purpura (HSP) and non-KD febrile illness (F) is highly variable but segregates from that of healthy children (HC). PCoA plot of beta diversity, based on Bray–Curtis dissimilarity between the genus-level profiles. A significant separation between patients and healthy controls was found (*p* = 0.002, permutation test with pseudo-F ratios). Ellipses include 95% confidence area based on the standard error of the weighted average of sample coordinates.

According to the PCoA of inter-individual variation, based on Bray–Curtis dissimilarity, the GM profiles of KD, HSP and F children were overall overlapped and spread out, suggesting high inter-individual variability. Despite this, all study groups segregated significantly from controls (*p* = 0.002, permutation test with pseudo-F ratios) (Figure 1).

With regard to the taxonomic composition (Supplementary Figure S2), the GM of all patients was dominated by the phylum Firmicutes (mean relative abundance in the whole cohort ± SD, 54.5% ± 13.7%), together with Bacteroidetes (17.3% ± 15.5%) and Actinobacteria (15.9% ± 12.7%). As expected for a child GM that is approximating the adult-like structure [38], the dominant families were *Lachnospiraceae* (15.5% ± 9.8%), *Ruminococcaceae* (10.6% ± 11.0%), *Bacteroidaceae* (10.8% ± 9.4%) and *Bifidobacteriaceae* (7.7% ± 9.5%). Consistently, *Bacteroides* (10.8% ± 9.4%), *Bifidobacterium* (7.7% ± 9.5%), *Streptococcus* (6.1% ± 12.6%) and unclassified members of *Lachnospiraceae* (7.1% ± 5.6%) and *Ruminococcaceae* (4.9% ± 7.3%) were the dominant genera.

**Figure 2:**
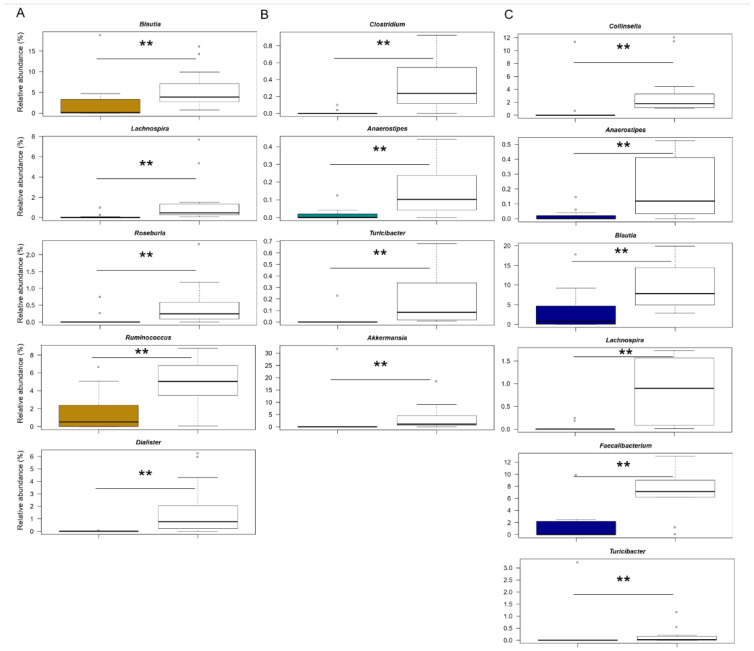
Compositional differences in the gut microbiota of patients with Kawasaki disease, Henoch–Schönlein purpura and non-KD febrile illness vs. healthy children. Boxplots showing the relative abundance distribution of bacterial genera significantly differentially represented between each patient group ((**A**) Kawasaki disease; (**B**) Henoch–Schönlein purpura; (**C**) non-KD febrile illness) and their respective controls, carefully matched by age and sex (same colour code as in Figure 1). Only taxa with relative abundance >0.1% in at least 3 samples are shown; ** for *p* < 0.01, Wilcoxon test.

When comparing GM structures between patient groups and their respective controls (carefully matched for age, a major microbiota-associated confounding factor) [39], common and disease-specific differences emerged (Figure 2).

Among the features shared by all patients, it is worth noting the underrepresentation of health-associated taxa, including *Lachnospiraceae* (e.g., *Anaerostipes*, *Lachnospira*, *Blautia* and *Roseburia*) and *Ruminococcaceae* (*Ruminococcus* and *Faecalibacterium*) genera (*p* ≤ 0.005, Wilcoxon test). Furthermore, both HSP and F children showed reduced proportions of *Turicibacter* (*p* ≤ 0.007). On the other hand, the underrepresentation of *Dialister* appeared to specifically characterise KD patients (*p* ≤ 0.001). As for HSP, the underrepresentation of *Clostridium* and *Akkermansia* was unique to this group of patients (*p* ≤ 0.005). Finally, a reduced relative abundance of *Collinsella* appeared to be specific to F (*p* ≤ 0.001). However, it should be noted that no significance was found for pairwise comparisons between children diagnosed with KD, HSP and F.

When searching for correlations with laboratory values, the relative abundance of *Blautia* was found to correlate negatively with CRP level (tau = −0.265, *p* = 0.03; Kendall rank correlation test) and a similar trend was observed for *Collinsella* (tau = −0.236, *p* = 0.08). Conversely, a direct correlation was found for *Akkermansia* (tau = 0.346, *p* = 0.009) (Supplementary Figure S3).

### 3.3. The Gut Microbiota Profiles of KD, HSP and F Children Stratify by Gastrointestinal Involvement

PCoA analysis based on Bray–Curtis distances between the genus-level profiles of all patients showed no segregation for variables known to be strongly associated with the GM composition [39], i.e., age (0–12 months, 1–5 years, 6–19 years), gender and ethnicity (Caucasian vs. others) (*p* > 0.3, permutation test with pseudo-F ratios) (Supplementary Figure S4).

**Figure 3:**
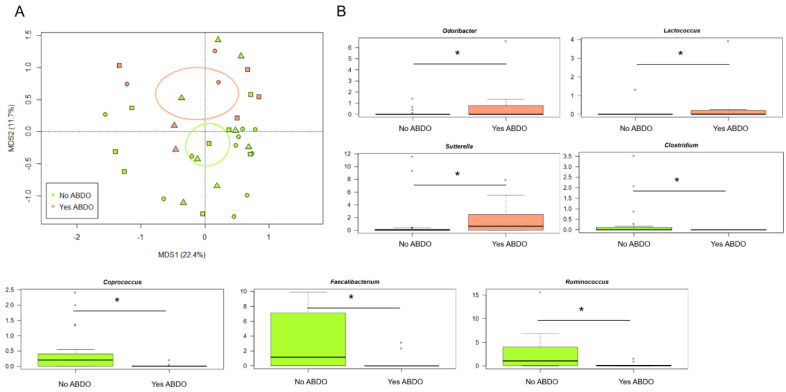
The gut microbiota profiles of patients with Kawasaki disease, Henoch–Schönlein purpura and non-KD febrile illness stratified by gastrointestinal involvement. (**A**) PCoA plot of beta diversity, based on Bray–Curtis dissimilarity between the genus-level profiles. A significant separation between patients with or without gastrointestinal involvement (ABDO 0 = no vs. ABDO 1 = yes) was found (*p* = 0.05, permutation test with pseudo-F ratios). Ellipses include 95% confidence area based on the standard error of the weighted average of sample coordinates. (**B**) Boxplots showing the relative abundance distribution of bacterial genera significantly differentially represented between groups (* *p* ≤ 0.05, post-hoc Wilcoxon test).

On the contrary, the GM structures of KD, HSP and F patients were found to stratify by abdominal involvement (ABDO 0 = no vs. ABDO 1 = yes) (*p* = 0.05) (Figure 3A). In particular, the genera *Odoribacter*, *Lactococcus* and *Sutterella* were overrepresented in children with abdominal involvement, while *Ruminococcus*, *Faecalibacterium*, *Coprococcus* and *Clostridium* were overrepresented in those without abdominal involvement (*p* ≤ 0.05, Wilcoxon test) (Figure 3B).

With specific regard to KD patients, no separation was observed between GM profiles in relation to clinical form (complete, i.e., with 4–5 signs vs. atypical/incomplete, i.e., with <4 signs) (*p* = 0.5, permutation test with pseudo-F ratios). On the other hand, a trend was found by response to treatment with intravenous immunoglobulin (IVIG) (IVIG-responders vs. IVIG non-responders) (*p* = 0.1) (Supplementary Figure S5).

Interestingly, in this cohort, IgA levels correlated positively with the relative abundance of *Bifidobacterium* (tau = 0.556, *p* = 0.04; Kendall rank correlation test) but negatively with that of *Klebsiella* (tau = −0.645, *p* = 0.03). Other significant inverse correlations were observed between calprotectin levels and proportions of unclassified genera of *Rikenellaceae*, as well as between IL12p70 and IL10 with *Blautia* and between TNF-alpha and *Christensenellaceae*_unclassified and *Butyricimonas* (tau ≤ −0.53, *p* ≤ 0.05). *Christensenellaceae*_unclassified also tended to correlate negatively with calprotectin (tau = −0.546, *p* = 0.06), but positively with IgG (tau = 0.546, *p* = 0.06). IgG levels tended to correlate positively with *Bifidobacterium* (tau = 0.5, *p* = 0.08) as well, but negatively with *Klebsiella* (tau = −0.559, *p* = 0.06). Other noteworthy trends included a direct correlation of TNF-alpha with *Eubacterium* (tau = 0.471, *p* = 0.09), and a negative one with *Coprococcus* and *Prevotella* (tau = −0.497 and −0.524, respectively; *p* = 0.08). Unclassified genera of *Lachnospiraceae* tended to correlate positively with IgA (tau = 0.5, *p* = 0.08) and IL10 (tau = 0.479, *p* = 0.07). Please see Supplementary Figure S6 for scatter plots of the correlation between genus-level relative abundances and inflammatory/immunological parameters.

## 4. Discussion

To our knowledge, this is the first study exploring the GM profile in a mostly Caucasian cohort of children diagnosed with HSP, KD and non-KD febrile condition.

Consistent with the existing literature on the GM of Asian subjects with KD and HSP [22,23,24,25], all study patients, compared to an age/gender-matched cohort of HC, showed some imbalances, including especially low proportions of typically health-associated and short-chain fatty acid (SCFA)-producing taxa, such as those belonging to the *Lachnospiraceae* and *Ruminococcaceae* families. However, it must be said that the decrease in SCFA producers is a common sign of dysbiosis, shared by various (enteric and non-enteric) diseases, possibly related to the presence of oxidative stress [40]. Although levels of SCFAs were not measured in the present study, their decrease could further affect the already compromised host immunological homeostasis [41]. Indeed, it has been shown that reduced levels of SCFAs induce Th17/Treg imbalances [42] and favour pathogen-derived hypercytokinemia, the latter being a potential environmental factor triggering KD development in genetically susceptible children [43]. Consistently, we documented expected correlations between known SCFA producers and pro/anti-inflammatory cytokines in KD, such as the inverse ones between *Blautia* and IL12p70, *Butyricimonas* and TNF-alpha and *Coprococcus* and TNF-alpha, and the positive one between *Lachnospiraceae* and IL10.

Interestingly, despite the small sample size, we were able to identify potential disease-specific candidates, i.e., *Dialister*, which was underrepresented in KD and *Clostridium* and *Akkermansia*, which were underrepresented in HSP. Furthermore, patients with F showed a low relative abundance of *Collinsella*. It should be noted that such potential signatures are different from those identified in previous studies [22,23,24,25], potentially being related to ethnicity and geographical effect, which are recognised as the main drivers of GM variation [27,44]. As for *Collinsella* and *Dialister*, they are genera typically represented in the infant GM as they are involved in the lactate cycle (lactose utiliser and lactate producer the first, lactate utiliser the second) [44,45]. Their underrepresentation in the KD and F groups, which included younger children, should be monitored over time, as it is known that early GM imbalances, even temporary, could have long-term repercussions on the child’s health [46,47,48]. Similarly, the reduced proportions of *Akkermansia* in children with HSP could be a red flag as this genus is important for gut and metabolic health, and can regulate immune responses [49,50,51].

Another interesting finding is that the GM structures of our patient population stratified by abdominal involvement. In particular, children with abdominal manifestations showed even smaller proportions of beneficial microbes (i.e., *Ruminococcus*, *Faecalibacterium*, *Coprococcus* and *Clostridium*) and increased amounts of the genera *Odoribacter*, *Lactococcus* and *Sutterella*. While no mechanistic links are available for these still poorly characterised microorganisms, it is worth mentioning that *Sutterella* was found to be prevalent in children with autism spectrum disorders with gastrointestinal dysfunction [52]. Intestinal involvement occurs in approximately one-half of HSP cases, ranging from mild to more significant and life-threatening findings [53]. Similarly, gastrointestinal involvement in KD is frequent [5] and has been correlated with a more severe form of the disease, characterised by coronary aneurysms [15]. Finally, with specific regard to KD, the GM profiles were found to be unrelated to the KD clinical features, but a trend towards separation was observed according to the IVIG response, suggesting a possible role of GM in modulating the host response, which deserves further investigation.

Our study has some strengths and some limitations. This is the first study comparing the most frequent vasculitis in childhood and non-KD febrile illness in a mostly Caucasian cohort. In addition, this is the first study exploring a possible association between GM and abdominal involvement in acute childhood illnesses. Furthermore, for the KD group, it was possible to correlate GM composition with pro/anti-inflammatory cytokines, and also faecal calprotectin, a common marker of intestinal inflammation [54]. The main limitations of this study are the small sample size, which is due to the low incidence of these vasculitis, especially KD, in our country, and the monocentric design of the study, which makes our data very preliminary. Furthermore, we found high inter-individual variability in GM composition, which likely prevented the detection of real GM-based signatures. Finally, although 16S rRNA amplicon sequencing is the gold standard for microbiome studies, it is a low-taxonomic resolution technique without the ability to provide functional information other than through inference.

In conclusion, GM alterations were described in our cohort of children with acute illnesses (KD, HSP, non-KD acute fever), with both shared and disease-specific features. The implications of these findings, although preliminary and based on a small cohort, are really promising: they could be a starting point for the design and introduction into clinical practice of potential GM-based therapies, in order to improve patient outcomes or modulate the disease severity. Further studies in larger cohorts, possibly including multiple ethnicities and prospectives to dissect the role of GM in the course of the disease, are urgently needed. For example, if the role of *Akkermansia* is confirmed, studies on barrier functionality will have to be conducted. Studies of this type, also employing other omics techniques (e.g., shotgun metagenomics, metatranscriptomics and metabolomics for high-resolution taxonomic and functional insights) and in vitro or animal models, could also contribute to the understanding of the etiopathogenesis of KD and HSP, both of which are still unclear.

## Figures and Tables

**Table 1:** Demographic, clinical and laboratory data of children diagnosed with Kawasaki disease (KD), Henoch–Schönlein purpura (HSP) and non-KD febrile illness (F).

		KD (n = 13)	HSP (n = 10)	F (n = 12)	*p*
Ethnicity, n (%)	Caucasian	10 (76.9%)	8 (80.0%)	8 (66.7%)	*n.s.*
	Asian	3 (23.1%)	2 (20.0%)	1 (8.3%)	
	Hispanic			1 (8.3%)	
	Mixed			1 (8.3%)	
	Black			1 (8.3%)	
Sex, n (%)	Male	10 (76.9%)	5 (50.0%)	7 (58.3%)	*n.s.*
	Female	3 (23.1%)	5 (50.0%)	5 (41.7%)	
Age (months), median (IQR)		31 (14.5–43) *	62 (52.3–104.3) *§	30 (15–67.8) §	0.005
Class, n (%)	Responder	11 (84.6%)	-	-	-
	Non-responder	2 (15.4%)			
Clinical form, n (%)	Complete	8 (61.5%)	-	-	-
	Atypical/ incomplete	5 (38.5%)			
Abdominal symptoms, n (%)	Yes	3 (23.1%)	2 (20.0%)	4 (33.3%)	*n.s.*
	No	10 (76.9%)	8 (80.0%)	8 (66.7%)	
White blood cells (×10^9^/L), median (IQR)		15.0 (12.3–20.0)	11.5 (8.7–15.2)	12.5 (10.8–13.7)	*n.s.*
Neutrophils %, median (IQR)		73.8 (59.7–80.3)	68.5 (58.1–73.5)	70 (59.9–76.6)	*n.s.*
Lymphocytes %, median (IQR)		19.0 (12.6–28.4)	28.6 (19.6–35.6)	20.6 (13.5–32.2)	*n.s.*
Eosinophils %, median (IQR)		1.4 (0.3–3.6)	1.7 (0.6–3.7)	0.5 (0.2–1.9)	*n.s.*
Red blood cells (×10^12^/L), median (IQR)		4.15 (3.90–4.46)	4.68 (4.12–4.97)	4.58 (4.01–5.79)	*n.s.*
Haemoglobin (g/dL), median (IQR)		10.7 (10.1–11.7) *	12.9 (11.0–13.8) *	11.2 (10.6–12.0)	0.034
Platelets count (×10^9^/L), median (IQR)		377 (317–536) °	358 (319–402)	293 (229–318) °	0.010
C-reactive protein (mg/dL), mean (SD)		9.68 (4.89) *	2.74 (3.11) *§	6.96 (4.29) §	0.002
Aspartate aminotransferase (IU/L), median (IQR)		32 (26–55)	29 (23–33)	36 (28–74)	*n.s.*
Alanine aminotransferase (IU/L), median (IQR)		27 (18–94) *	14 (9–16) *	16 (10–25)	0.011
Calprotectin (mcg/g), median (IQR)		706 (117–1445)	-	175 (38–501)	*n.s.*
TNF-alpha (pg/mL), median (IQR)		2.5 (0–24.3)	-	1.0 (0–2.8)	*n.s.*
IL6 (pg/mL), median (IQR)		132.0 (58.4–1747.0)	-	18.1 (8.6–104.5)	0.025
IL8 (pg/mL), median (IQR)		214 (62–14910)	-	123 (18–4502)	*n.s.*
IL12p70 (pg/mL), median (IQR)		0 (0–5)	-	0 (0–1.25)	*n.s.*
IL10 (pg/mL), median (IQR)		7.0 (1.8–30.3)	-	6.0 (3.3–16.0)	*n.s.*
IgG (mg/dL), median (IQR)		729 (664–861)	1044 (850–1237)	944 (674–1299)	*n.s.*
IgM (mg/dL), mean (SD)		96.6 (48.2)	102.0 (44.2)	111.6 (29.5)	*n.s.*
IgA (mg/dL), mean (SD)		101.5 (80.3) *	170.2 (65.2) *§	96.3 (51.6) §	0.031
Cardiac non-coronary involvement, n (%)	Yes	7 (53.8%)	-	-	-
	No	6 (46.2%)			
Coronary involvement, n (%)	Yes	7 (53.8%)	-	-	-
	No	6 (46.2%)			
Coronary artery lesions, n (%)	No involvement	6 (46.2%)	-	-	-
	Dilations	2 (15.4%)			
	Aneurysms	5 (38.5%)			
Total days of fever, median (IQR)		8 (7–10)	-	8 (6–10)	*n.s.*
Delayed therapy, n (%)	Yes	2 (15.4%)	-	-	-
	No	10 (76.9%)			

*n.s.* stands for “not significant”; * stands for statistically significant difference between KD and HSP; § stands for statistically significant difference between HSP and F; ° stands for statistically significant difference between KD and F.

## Data Availability

Sequencing reads were deposited in the National Center for Biotechnology Information Sequence Read Archive (NCBI SRA; BioProject ID PRJNA807281). The name of the repository and accession number can be found in the article.

## Supplementary Materials

The following supporting information can be downloaded at: https://www.mdpi.com/article/10.3390/jpm12060973/s1, Figure S1: Alpha diversity of the gut microbiota in paediatric patients with Kawasaki disease, Henoch–Schönlein purpura and non-KD febrile illness vs. healthy children; Figure S2: Gut microbiota structure at phylum and family level in paediatric patients with Kawasaki disease, Henoch–Schönlein purpura and non-KD febrile illness vs. healthy children; Figure S3: Associations between genus-level relative abundances and levels of C-reactive protein in paediatric patients with Kawasaki disease, Henoch–Schönlein purpura and non-KD febrile illness; Figure S4: The GM dysbiosis in paediatric patients with Kawasaki disease, Henoch–Schönlein purpura and non-KD febrile illness is independent of potential confounding factors; Figure S5: The gut microbiota profiles of paediatric patients with Kawasaki disease tend to stratify by response to therapy; Figure S6: Associations between genus-level relative abundances and levels of inflammatory/immunological parameters in paediatric patients with Kawasaki disease.

## References

[1] Weiss P.F. (2012). Pediatric vasculitis. Pediatrics Clin. N. Am..

[2] Bowyer S., Roettcher P. (1996). Pediatric rheumatology clinic populations in the United States: Results of a 3 year survey. J. Rheumatol..

[3] Hetland L.E., Susrud K.S., Lindahl K.H., Bygum A. (2017). Henoch-Schönlein Purpura: A Literature Review. Acta Derm. Venereol..

[4] Ozen S., Pistorio A., Iusan S.M., Bakkaloglu A., Herlin T., Brik R., Buoncompagni A., Lazar C., Bilge I., Uziel Y. (2010). EULAR/PRINTO/PRES criteria for Henoch–Schönlein purpura, childhood polyarteritis nodosa, childhood Wegener granulomatosis and childhood Takayasu arteritis: Ankara 2008. Part II: Final classification criteria. Ann. Rheum. Dis..

[5] McCrindle B.W., Rowley A.H., Newburger J.W., Burns J.C., Bolger A.F., Gewitz M., Baker A.L., Jackson M.A., Takahashi M., Shah P.B. (2017). Diagnosis, treatment, and long-term management of Kawasaki disease: A scientific statement for health professionals from the American Heart Association. Circulation.

[6] Rowley A., Baker S.C., Arrollo D., Gruen L.J., Bodnar T., Innocentini N., Hackbart M., Pulido Y.C., Wylie K.M., A Kim K.-Y. (2020). A protein epitope targeted by the antibody response to Kawasaki disease. J. Infect. Dis..

[7] Rowley A.H., Baker S.C., Shulman S.T., Rand K.H., Tretiakova M.S., Perlman E., Garcia F.L., Tajuddin N.F., Fox L.M., Huang J.H. (2011). Ultrastructural, immunofluorescence, and RNA evidence support the hypothesis of a “new” virus associated with Kawasaki disease. J. Infect. Dis..

[8] Rowley A.H., Baker S.C., Shulman S.T., Garcia F.L., Fox L.M., Kos I.M., Crawford S.E., Russo P.A., Hammadeh R., Takahashi K. (2008). RNA-containing cytoplasmic inclusion bodies in ciliated bronchial epithelium months to years after acute Kawasaki disease. PLoS ONE.

[9] Yim D., Curtis N., Cheung M., Burgner D. (2013). Update on Kawasaki disease: Epidemiology, aetiology and pathogenesis. J. Paediatr. Child. Health.

[10] Takeshita S., Nakatani K., Kawase H., Seki S., Yamamoto M., Sekine I., Yoshioka S. (1999). The role of bacterial lipopolysaccharide-bound neutrophils in the pathogenesis of Kawasaki disease. J. Infect. Dis..

[11] Kim G.B. (2019). Reality of Kawasaki disease epidemiology. Korean J. Pediatrics.

[12] Makino N., Nakamura Y., Yashiro M., Kosami K., Matsubara Y., Ae R., Aoyama Y., Yanagawa H. (2019). Nationwide epidemiologic survey of Kawasaki disease in Japan, 2015–2016. Pediatrics Int..

[13] Mauro A., Fabi M., Da Frè M., Guastaroba P., Corinaldesi E., Calabri G.B., Giani T., Simonini G., Rusconi F., Cimaz R. (2016). Kawasaki disease: An epidemiological study in central Italy. Pediatrics Rheumatol. Online J..

[14] Carmona E.G., García-Giménez J.A., López-Mejías R., Khor C.C., Lee J.K., Taskiran E., Ozen S., Hocevar A., Liu L., Gorenjak M. (2021). Identification of a shared genetic risk locus for Kawasaki disease and IgA vasculitis by a cross-phenotype meta-analysis. Rheumatology.

[15] Fabi M., Corinaldesi E., Pierantoni L., Mazzoni E., Landini C., Bigucci B., Ancora G., Malaigia L., Bodnar T., Di Fazzio G. (2018). Gastrointestinal presentation of Kawasaki disease: A red flag for severe disease?. PLoS ONE.

[16] Turroni S., Brigidi P., Cavalli A., Candela M. (2018). Microbiota-host transgenomic metabolism, bioactive molecules from the inside: Miniperspective. J. Med. Chem..

[17] Zheng D., Liwinski T., Elinav E. (2020). Interaction between microbiota and immunity in health and disease. Cell Res..

[18] Ling Z., Li Z., Liu X., Cheng Y., Luo Y., Tong X., Yuan L., Wang Y., Sun J., Li L. (2014). Altered Fecal Microbiota Composition Associated with Food Allergy in Infants. Appl. Environ. Microbiol..

[19] Cheng J., Palva A.M., Vos W.M.D., Satokari R. (2013). Contribution of the Intestinal Microbiota to Human Health: From Birth to 100 Years of Age. Curr. Top. Microbiol. Immunol..

[20] Wu H.J., Wu E. (2012). The role of gut microbiota in immune homeostasis and autoimmunity. Gut Microbes.

[21] Berer K., Mues M., Koutrolos M., Al Rasbi Z., Boziki M., Johner C., Wekerle H., Krishnamoorthy G. (2011). Commensal microbiota and myelin autoantigen cooperate to trigger autoimmune demyelination. Nature.

[22] Shen J., Ding Y., Yang Z., Zhang X., Zhao M. (2020). Effects of changes on gut microbiota in children with acute Kawasaki disease. PeerJ.

[23] Chen J., Yue Y., Wang L., Deng Z., Yuan Y., Zhao M., Yuan Z., Tan C., Cao Y. (2020). Altered gut microbiota correlated with systemic inflammation in children with Kawasaki disease. Sci. Rep..

[24] Wang X., Zhang L., Wang Y., Liu X., Zhang H., Liu Y., Shen N., Yang J., Gai Z. (2018). Gut microbiota dysbiosis is associated with Henoch-Schönlein Purpura in children. Int. Immunopharmacol..

[25] Kinumaki A., Sekizuka T., Hamada H., Kato K., Yamashita A., Kuroda M. (2015). Characterization of the gut microbiota of Kawasaki disease patients by metagenomic analysis. Front. Microbiol..

[26] Nagelkerke S.Q., Tacke C.E., Breunis W.B., Tanck M.W.T., Geissler J., Png E., Hoang L.T., van der Heijden J., Naim A.N.M., Yeung R.S.M. (2019). Extensive Ethnic Variation and Linkage Disequilibrium at the FCGR2/3 Locus: Different Genetic Associations Revealed in Kawasaki Disease. Front. Immunol..

[27] Deschasaux M., Bouter K.E., Prodan A., Levin E., Groen A.K., Herrema H., Tremaroli V., Bakker G.J., Attaye I., Pinto-Sietsma S.-J. (2018). Depicting the composition of gut microbiota in a population with varied ethnic origins but shared geography. Nat. Med..

[28] Cancello R., Turroni S., Rampelli S., Cattaldo S., Candela M., Cattani L., Mai S., Vietti R., Scacchi M., Brigidi P. (2019). Effect of short-term dietary intervention and probiotic mix supplementation on the gut microbiota of elderly obese women. Nutrients.

[29] D’Amico F., Biagi E., Rampelli S., Fiori J., Zama D., Soverini M., Barone M., Leardini D., Muratore E., Prete A. (2019). Enteral nutrition in pediatric patients undergoing hematopoietic SCT promotes the recovery of gut microbiome homeostasis. Nutrients.

[30] Masella A.P., Bartram A.K., Truszkowski J.M., Brown D.G., Neufeld J.D. (2012). PANDAseq: Paired-end assembler for illumina sequences. BMC Bioinform..

[31] Bolyen E., Rideout J.R., Dillon M.R., Bokulich N.A., Abnet C.C., Al-Ghalith G.A., Alexander H., Alm E.J., Arumugam M., Asnicar F. (2018). QIIME 2: Reproducible, interactive, scalable, and extensible microbiome data science. Nat. Biotechnol..

[32] Callahan B.J., McMurdie P.J., Rosen M.J., Han A.W., Johnson A.J., Holmes S.P. (2016). DADA2: High-resolution sample inference from Illumina amplicon data. Nat. Methods.

[33] Rognes T., Flouri T., Nichols B., Quince C., Mahé F. (2016). VSEARCH: A versatile open source tool for metagenomics. PeerJ.

[34] Rampelli S., Guenther K., Turroni S., Wolters M., Veidebaum T., Kourides Y., Molnár D., Lissner L., Benitez-Paez A., Sanz Y. (2018). Pre-obese children’s dysbiotic gut microbiome and unhealthy diets may predict the development of obesity. Commun. Biol..

[35] Muleviciene A., D’Amico F., Turroni S., Candela M., Jankauskiene A. (2018). Iron deficiency anemia-related gut microbiota dysbiosis in infants and young children: A pilot study. Acta Microbiol. Immunol. Hung..

[36] Biagi E., Franceschi C., Rampelli S., Severgnini M., Ostan R., Turroni S., Consolandi C., Quercia S., Scurti M., Monti D. (2016). Gut microbiota and extreme longevity. Curr. Biol..

[37] Culhane A.C., Thioulouse J., Perrière G., Higgins D.G. (2005). MADE4: An R package for multivariate analysis of gene expression data. Bioinformatics.

[38] Derrien M., Alvarez A.S., de Vos W.M. (2019). The gut microbiota in the first decade of life. Trends Microbiol..

[39] Vujkovic-Cvijin I., Sklar J., Jiang L., Natarajan L., Knight R., Belkaid Y. (2020). Host variables confound gut microbiota studies of human disease. Nature.

[40] Duvallet C., Gibbons S., Gurry T., Irizarry R., Alm E. (2017). Meta-analysis of gut microbiome studies identifies disease-specific and shared responses. Nat. Commun..

[41] Koh A., De Vadder F., Kovatcheva-Datchary P., Bäckhed F. (2016). From dietary fiber to host physiology: Short-chain fatty acids as key bacterial metabolites. Cell.

[42] Kaneko K., Akagawa S., Akagawa Y., Kimata T., Tsuji S. (2020). Our evolving understanding of Kawasaki disease pathogenesis: Role of the gut microbiota. Front. Immunol..

[43] Nakamura A., Ikeda K., Hamaoka K. (2019). Aetiological significance of infectious stimuli in Kawasaki disease. Front. Pediatrics.

[44] Bäckhed F., Roswall J., Peng Y., Feng Q., Jia H., Kovatcheva-Datchary P., Li Y., Xia Y., Xie H., Zhong H. (2015). Dynamics and stabilization of the human gut microbiome during the first year of life. Cell Host Microbe.

[45] Milani C., Duranti S., Bottacini F., Casey E., Turroni F., Mahony J., Belzer C., Delgado Palacio S., Arboleya Montes S., Mancabelli L. (2017). The first microbial colonizers of the human gut: Composition, activities, and health implications of the infant gut microbiota. Microbiol. Mol. Biol. Rev..

[46] Stanislawski M.A., Dabelea D., Wagner B.D., Iszatt N., Dahl C., Sontag M.K., Knight R., Lozupone C.A., Eggesbø M. (2018). Gut microbiota in the first 2 years of life and the association with body mass index at age 12 in a Norwegian birth cohort. mBio.

[47] Ho N.T., Li F., Lee-Sarwar K.A., Tun H.M., Brown B., Pannaraj P.S., Bender J.M., Azad M.B., Thompson A.L., Weiss S.T. (2018). Meta-analysis of effects of exclusive breastfeeding on infant gut microbiota across populations. Nat. Commun..

[48] Stokholm J., Blaser M.J., Thorsen J., Rasmussen M.A., Waage J., Vinding R.K., Schoos A.-M.M., Kunøe A., Fink N.R., Chawes B. (2018). Maturation of the gut microbiome and risk of asthma in childhood. Nat. Commun..

[49] Everard A., Belzer C., Geurts L., Ouwerkerk J.P., Druart C., Bindels L.B., Guiot Y., Derrien M., Muccioli G.G., Delzenne N.M. (2013). Cross-talk between Akkermansia muciniphila and intestinal epithelium controls diet-induced obesity. Proc. Natl. Acad. Sci. USA.

[50] De Vos W.M. (2017). Microbe Profile: Akkermansia muciniphila: A conserved intestinal symbiont that acts as the gatekeeper of our mucosa. Microbiology.

[51] Derrien M., Van Baarlen P., Hooiveld G., Norin E., Muller M., de Vos W. (2011). Modulation of mucosal immune response, tolerance, and proliferation in mice colonized by the mucin-degrader Akkermansia muciniphila. Front. Microbiol..

[52] Williams B.L., Hornig M., Parekh T., Lipkin W.I. (2012). Application of novel PCR-based methods for detection, quantitation, and phylogenetic characterization of Sutterella species in intestinal biopsy samples from children with autism and gastrointestinal disturbances. mBio.

[53] Chang W.L., Yang Y.H., Lin Y.T., Chiang B.L. (2004). Gastrointestinal manifestations in Henoch-Schönlein purpura: A review of 261 patients. Acta Paediatrics.

[54] Mosli M., Zou G., Garg S.K., Feagan S.G., MacDonald J.K., Chande N., Sandborn W.J., Feagan B.G. (2015). C-reactive protein, fecal calprotectin, and stool lactoferrin for detection of endoscopic activity in symptomatic inflammatory bowel disease patients: A systematic review and meta-analysis. Am. J. Gastroenterol..

