# Supplementary material for: Gut Microbiota Dysbiosis in Childhood Vasculitis: A Perspective Comparative Pilot Study
Source: J Pers Med. 2022 Jun 15;12(6):973. doi: 10.3390/jpm12060973 (PMC9224684; doi:10.3390/jpm12060973)
Supplement: Supplementary file 1 [file jpm-12-00973-s001.zip › jpm-1731594-supplementary.pdf]

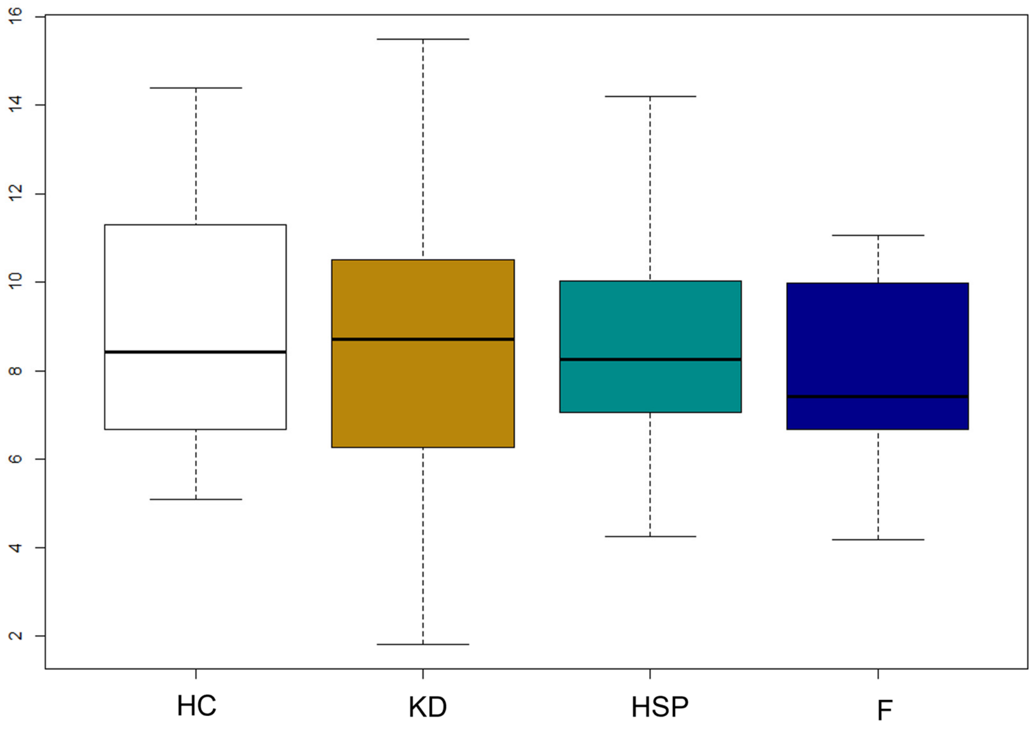

Figure S1. Alpha diversity of the gut microbiota in paediatric patients with Kawasaki disease, Henoch–Schönlein purpura and non-KD febrile illness vs. healthy children.

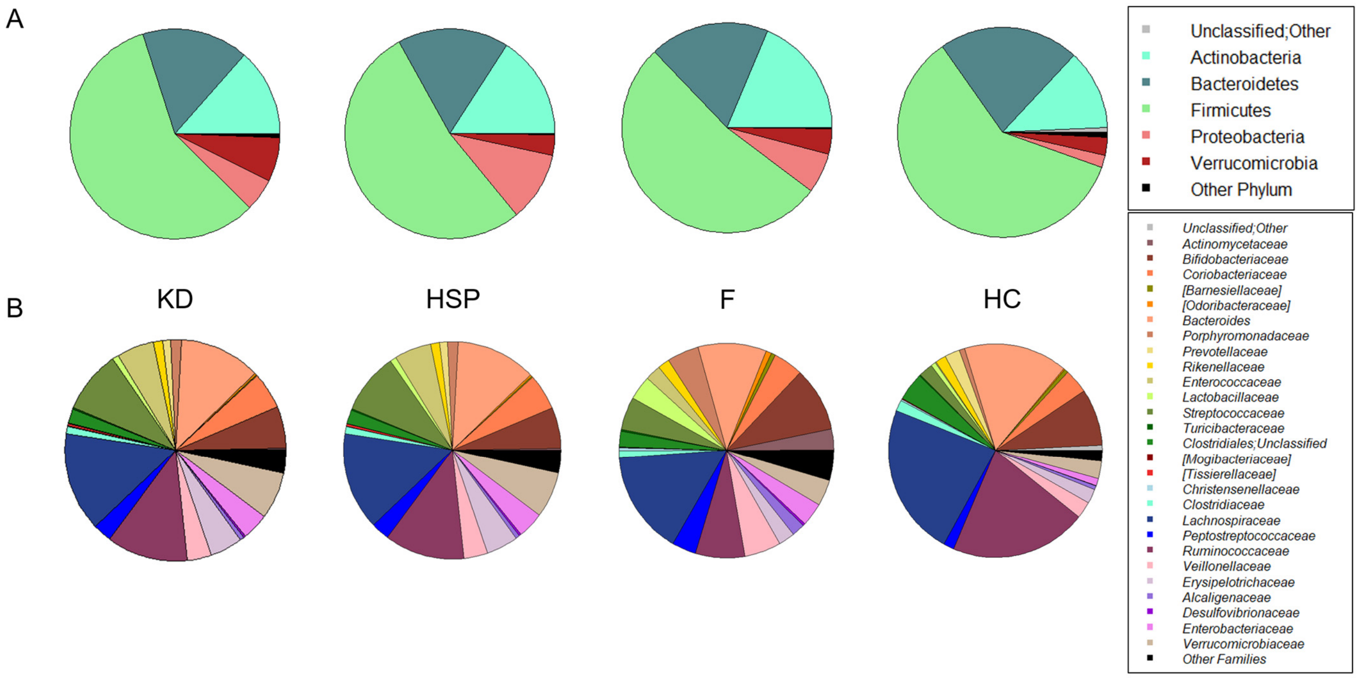

Figure S2. Gut microbiota structure at phylum and family level in paediatric patients with Kawasaki disease, Henoch–Schönlein purpura and non-KD febrile illness vs. healthy children.

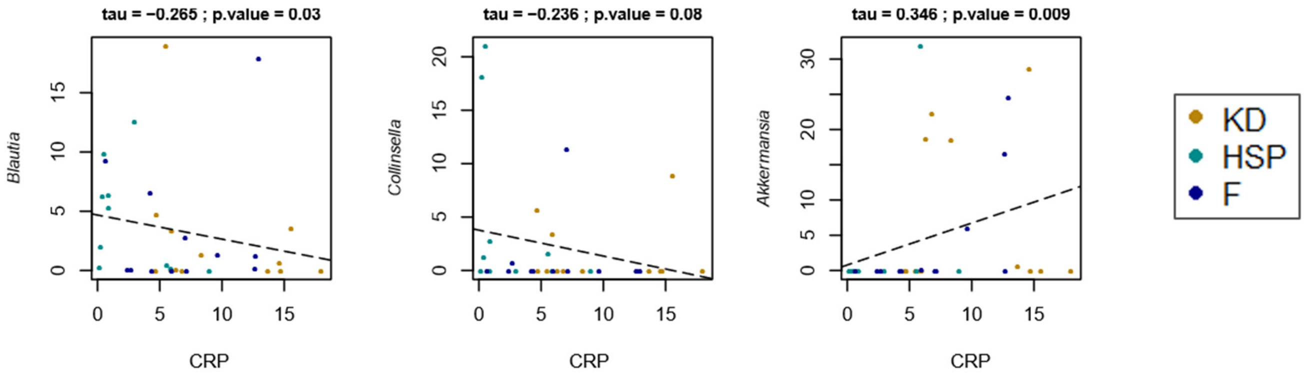

Figure S3. Associations between genus-level relative abundances and levels of C-reactive protein in paediatric patients with Kawasaki disease, Henoch–Schönlein purpura and non-KD febrile illness.

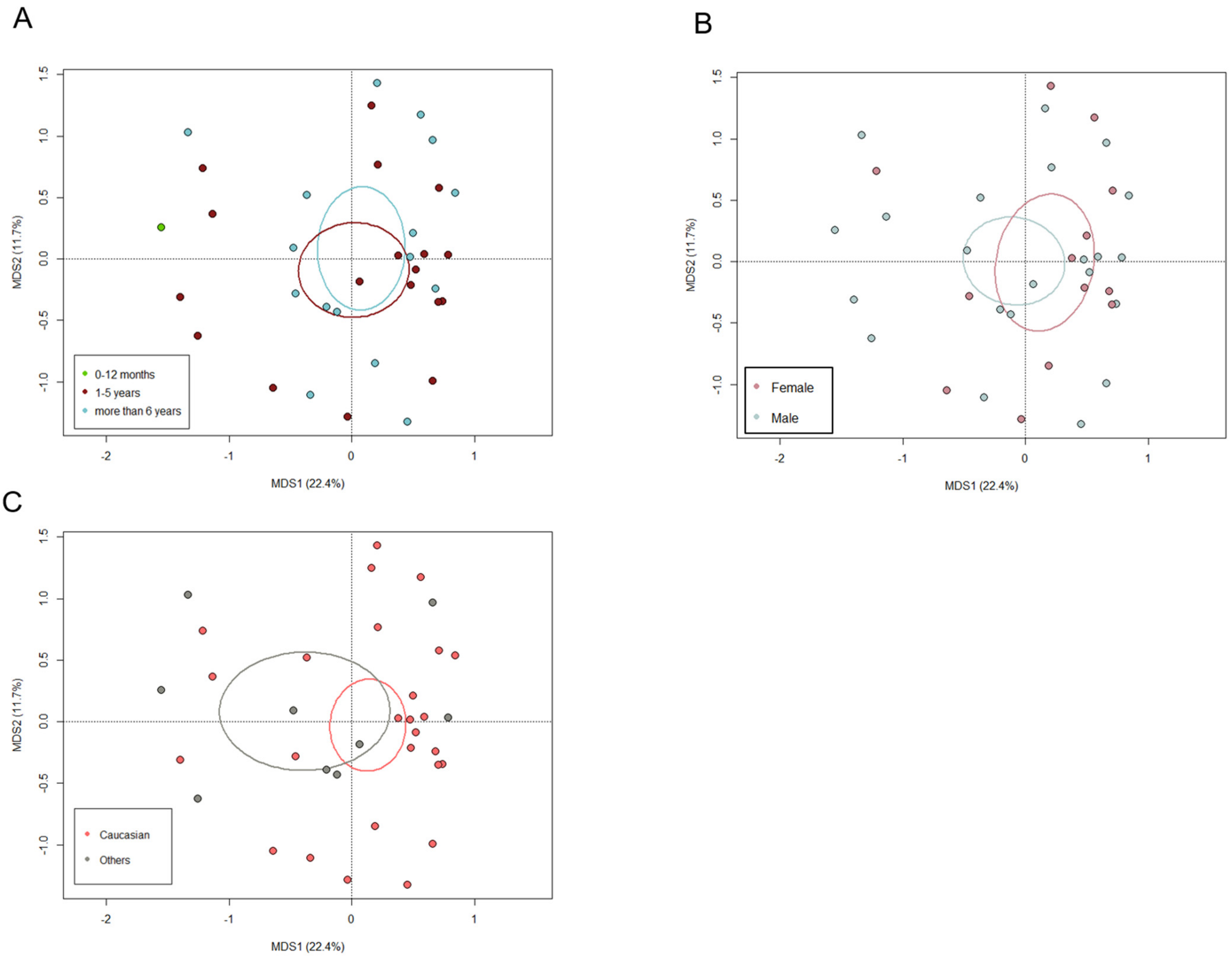

Figure S4. The GM dysbiosis in paediatric patients with Kawasaki disease, Henoch–Schönlein purpura and non-KD febrile illness is independent of potential confounding factors.

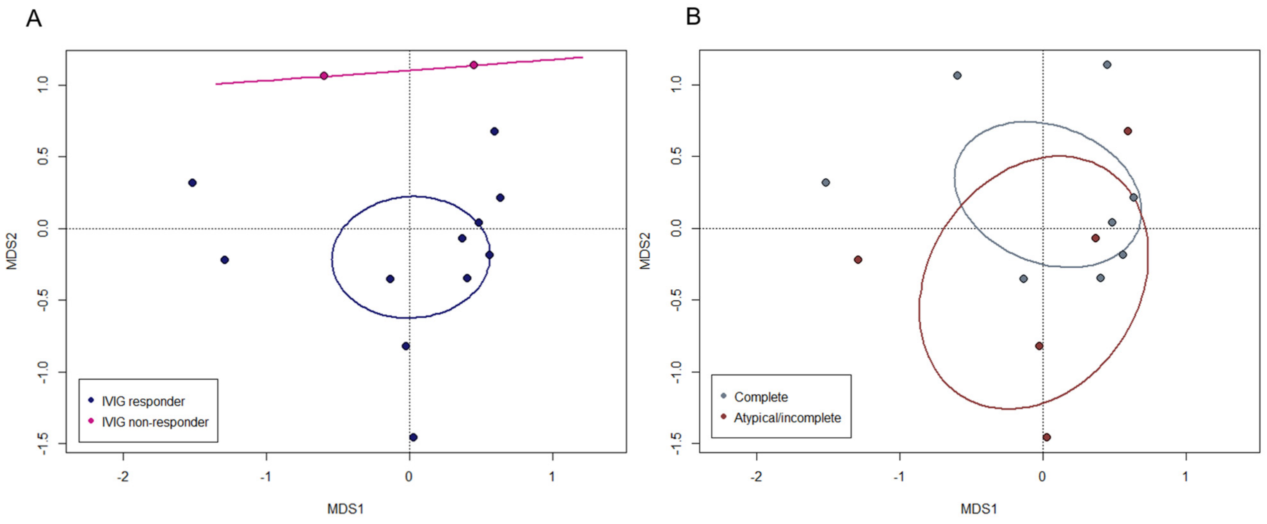

Figure S5. The gut microbiota profiles of paediatric patients with Kawasaki disease tend to stratify by response to therapy.

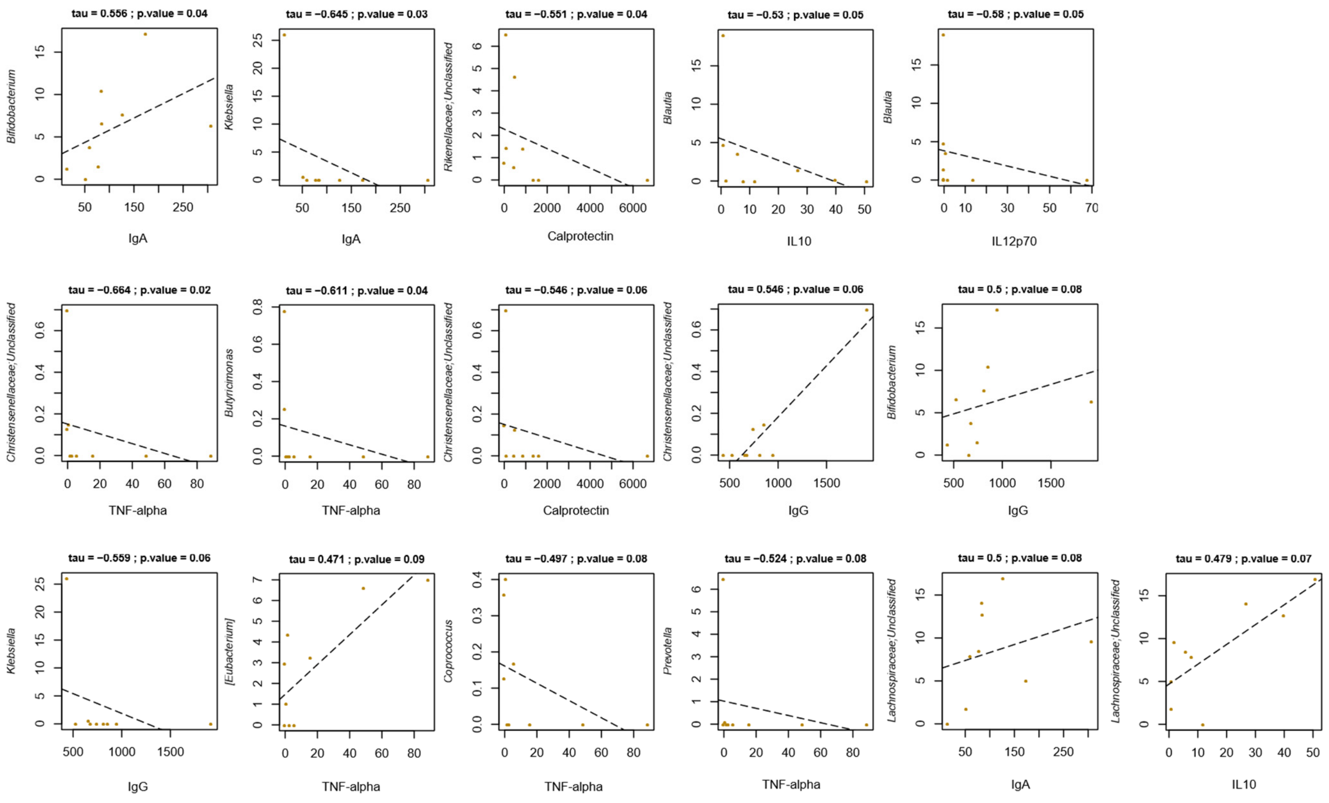

Figure S6. Associations between genus-level relative abundances and levels of inflammatory/immunological parameters in paediatric patients with Kawasaki disease.
